# Comparative proteome analysis of abdominal adipose tissues between fat and lean broilers

**DOI:** 10.1186/s12953-016-0100-2

**Published:** 2016-09-01

**Authors:** Chun-Yan Wu, Yuan-Yuan Wu, Chun-Dong Liu, Yu-Xiang Wang, Wei Na, Ning Wang, Hui Li

**Affiliations:** 1 Key Laboratory of Chicken Genetics and Breeding of Agriculture Ministry, Key Laboratory of Animal Genetics, Breeding and Reproduction of Education Department of Heilongjiang Province, College of Animal Science and Technology, Northeast Agricultural University, Harbin, 150030 Heilongjiang China; 2 Weifang Academy of Agricultural Sciences, Weifang, 261071 Shandong China

**Keywords:** Abdominal adipose tissue, Broiler, Proteomics, Differentially expressed protein

## Abstract

**Background:**

The molecular mechanism underlying broiler fat deposition is still poorly understood.

**Method:**

Currently, we used two-dimensional gel electrophoresis (2DE) to identify differentially expressed proteins in abdominal adipose tissues of birds at 4 week of age derived from Northeast Agricultural University broiler lines divergently selected for abdominal fat content (NEAUHLF).

**Results:**

Thirteen differentially expressed protein spots were screened out and identified by matrix-assisted laser desorption-ionization time-of-flight mass spectrometry (MALDI-TOF-MS). The protein spots were matched to thirteen proteins by searching against the NCBInr database. These identified proteins were apolipoprotein A-I (Apo A-I), cytokeratin otokeratin, ATP synthase subunit alpha, peptidyl-prolyl cis-trans isomerase FKBP4 (PPIase FKBP4), aspartate aminotransferase, carbonic anhydrase II (CA-II), prostaglandin-H2 D-isomerase precursor, fibrinogen alpha chain, lamin-A (LMNA), superoxide dismutase [Mn] (MnSOD), heat shock protein beta-1 (HSPβ1) and two predicted proteins. These differentially expressed proteins are involved mainly in lipid metabolism, amino acid metabolism, signal transduction, energy conversion, antioxidant, and cytoskeleton. Differential expression of Apo A-I, PPIase FKBP4, and cytokeratin otokeratin proteins were further confirmed by Western blot analysis. Quantitative real-time RT-PCR analyses showed that, of these 13 differentially expressed proteins, only PPIase FKBP4 and cytokeratin otokeratin were differentially expressed at mRNA level between the two lines.

**Conclusions:**

Our results have provided further information for understanding the basic genetics control of growth and development of broiler adipose tissue.

## Background

Great progress has been made in poultry breeding in the past half century. Daily gain and feed conversion have been improved considerably; however, in commercial flocks, the improved productive performance is accompanied by high percentages of body fat content and some other negative effects that bring huge economic losses to the broiler industry [1]. Controlling fat deposition has been one of the major goals in the broiler breeding industry.

Adipose tissue not only serves as a fat storage site, but also as an endocrine organ that plays roles in a wide range of cellular processes including lipid metabolism and glucose homeostasis [2]. In chicken, abdominal adipose tissue is the main tissue of body fat accumulation, accounting for about 22 % of total body fat [3]. To control broiler fat deposition, it is necessary to understand gene expression and its regulation during adipose tissue development. Gene expression profiling of chicken abdominal adipose tissue has been performed, and a number of differentially expressed genes have been identified between fat and lean chickens [4–8]. With the advent of proteomic technologies, comprehensive proteomic approaches have been widely used to identify and relatively quantify proteins.

In the present study, we compared protein expression profiles of abdominal adipose tissues of birds at 4 week of age derived from NEAUHLF, and found 13 differentially expressed proteins between fat and lean broilers. Our findings provide further information for understanding the molecular mechanism of broiler fat deposition.

## Methods

### Animal and abdominal adipose tissue samples collection

The NEAUHLF [9] was used in the current study. All animal work was conducted on the basis of the guidelines for the care and use of experimental animals established by the Ministry of Science and Technology of the People’s Republic of China (Approval number: 2006-398) and approved by the Laboratory Animal Management Committee of Northeast Agricultural University. All broilers were kept in similar environmental conditions and had free access to feed and water. Commercial corn-soybean-based diets, which met all the NRC requirements were provided to the broilers [10].

Six male broilers from the 13^th^ generation of NEAUHLF, three from the lean line and three from the fat line, were used in the present study. The average abdominal fat percentage of the three fat broilers was 9.34 times greater than that of the three lean broilers (Table 1). The broilers were slaughtered at 4 weeks of age, and abdominal adipose tissues were collected, frozen immediately in liquid nitrogen, and stored at -80 °C until further use.

**Table 1:** Body weight (BW), abdominal fat weigh (AFW) and abdominal fat percentage (AFP) of the fat and lean broilers at 4 weeks of age

Traits	Lean line			Fat line		
	Lean 1	Lean 2	Lean 3	Fat 1	Fat 2	Fat 3
BW (g)	727.50	726.80	741.30	783.50	662.10	853.80
AFW (g)	3.65	3.59	3.85	41.27	30.91	31.44
AFP (%)	0.50	0.49	0.51	5.30	4.60	4.10

Lean 1, Lean 2, Lean 3 were three lean broilers; Fat 1, Fat 2, Fat 3 were three fat broilers

### Protein samples preparation for 2DE

Total protein was isolated from the abdominal adipose tissues by Trizol reagent (Invitrogen, Carlsbad, CA, USA) according to the manufacturer’s protocol with minor modifications [11]. The samples were then dissolved in lysis buffer containing 8 M urea, 2 M thiourea, 4 % (wt/vol) 3-[(3-cholamidopropyl) dimethylammonio]-1-propanesulfonate, 2 % carrier ampholytes (pH 3 to 10, GE Healthcare, Uppsala, Sweden), 50 mM dithiothreitol (DTT), and 1× protease inhibitor cocktails (Roche Diagnostics GmbH, Mannheim, Germany). The original protein samples were centrifuged at 20,000 g for 1 h to remove insoluble materials. For 2DE, salt and other small molecular impurities were removed using the 2D Clean-Up Kit (GE Healthcare, Chalfont St Giles, UK). Total protein concentration was determined using a 2D Quant Kit (Amersham Biosciences Corp., Piscataway, NJ, USA) and the protein samples were then stored at -80 °C.

### 2DE and image analyses

Protein samples were rehydrated at 650 μg/gel in 350 μL of rehydration solution containing 7 M urea, 2 M thiourea, 4 % (wt/vol) 3-[(3-cholamidopropyl) dimethylammonio]-1-propanesulfonate, 50 mM DTT and 0.8 % carrier ampholytes (pH 3 to 10, GE Healthcare). First-dimension electrophoresis was conducted with the IPGphor3 isoelectric focusing system (GE Healthcare) using the dry IPG strips (18 cm pH 3 to 10 nonlinear, GE Healthcare). The program setting was as follows: 50 V for 12 h, 100 V for 1 h, 300 V for 1 h, linear gradient to 1000 V in 2.5 h, linear gradient to 8000 V in 2 h and 8000 V until approximately 60,000 Vh. After the first dimension, the IPG strips were equilibrated in SDS equilibration buffer containing 75 mM Tris-HCl, pH 8.8, 6 M urea, 30 % (v/v) glycerol, 2 % (w/v) SDS, bromophenol blue 1 % (w/v) for 15 min, followed by a second equilibration with 2.5 % iodoacetamide replacing the 2 % DTT for 15 min. After equilibration, the proteins were separated on 12.5 % Trisglycine gels using an Ettan Dalt Six Electrophoresis System (GE Healthcare) at 12 °C. Gels were run at constant power, first with two W/strip for 45 min and then 15 W/strip until the bromophenol blue reached the bottom of the gels. Then, the gels were stained by the blue silver method with coomassie blue brilliant G250 [12]. Finally, six 2-DE gels were obtained.

Protein spot detection, volume calculation, matching, and the patterns were analyzed using Image Master 2D Platinum 6.0 software (GE Healthcare). The parameter used for the quantifications was the % of volume (%VOL: integration of the OD over the feature area (VOL) normalized by the total VOL over the whole image). Differentially expressed protein spots were considered significant if they showed >2-fold relative differences (*P* <0.05, Student’s *t* test) between the fat and lean lines.

### Protein identification by MALDI-TOF-MS

After image analyses, the differentially expressed protein spots were selected and excised from the gels. The protein spots were subjected to tryptic proteolysis, and the resultant peptides were analyzed by matrix-assisted laser desorption- ionization time-of-flight mass spectrometry (MALDI-TOF-MS) as described previously [13]. The resultant peptide mass fingerprint was searched against the NCBInr protein sequence databases using the Mascot search engine [14]. The search parameters were as follows: enzyme search specificity was trypsin for tryptic digest; carbamidomethylation on cysteines was set as fixed modification while methionine oxidation was considered as variable modification; one miscleavage for each peptide was allowed; no restrictions on protein mass and peptide mass tolerance was ±100 ppm. A Mascot score with *P* <0.05 was considered statistically significant [15].

### Western blot analysis

The abdominal adipose tissue was homogenized in radio immunoprecipitation assay (RIPA) buffer (1 g/L SDS, 5 g/L sodium deoxycholate, 10 g/L Nonidet P-40, 150 mmol/L NaCl, 50 mmol/L Tris-HCl, pH 8.0), supplemented with protease inhibitors (1 mmol/L phenylmethylsulfonyl fluoride, 0.002 g/L aprotinin and 0.002 g/L leupeptin). Cellular debris and lipids were eliminated by centrifuging the solubilized samples at 13,000 rpm for 60 min. The protein concentration of the samples was determined using a 2D Quant kit.

Protein samples were separated by SDS-PAGE and transferred to an Immun-Blot PVDF membrane (Millipore, Billerica, MA, USA). To block nonspecific binding, the membrane was incubated in blocking buffer (PBS with 5 % nonfat dry milk) for 1 h at room temperature. Membranes were immunoblotted with antibodies against Apo A-I (BIOSS, Beijing, China; 1:500 dilution), PPIase FKBP4 (ProteinTech Group, Chicago, IL, USA; 1: 500 dilution), and cytokeratin otokeratin (ProteinTech Group, Chicago, IL, USA; 1: 500 dilution) for 1 h at room temperature. After washing with PBS with 0.05 % Tween-20 (PBST), the membrane was immunoblotted with goat anti-rabbit IgG conjugated with horseradish peroxidase (1:5000) (ZSGB-BIO, Beijing, China) for 1 h at room temperature. Immunoreactive protein on the membrane was visualized using enhanced chemiluminescence and exposed to X-ray-film (Kodak, New York, NY, USA). β-actin (as the control) was detected first by mouse anti-chicken (β-actin) antibody (Beyotime Institute of Biotechnology, Jiangsu, China) and then by peroxidase-conjugated AffiniPure goat anti-mouse IgG (H + L; ZSGB-Bio). Immunoreactive protein levels were determined semi-quantitatively by densitometric analysis using the UVP system Labworks TM software 3.0 (UVP, Upland, CA, USA). Results were expressed as the relative quantity of Apo A-I/β-actin, PPIase FKBP4/β-actin and cytokeratin otokeratin/β-actin.

### Real-time RT-PCR analyses

Total RNA from abdominal adipose tissue was isolated using Trizol reagent. Reverse transcription was performed using 1 μg of total RNA and M-MLV reverse transcriptase (Moloney murine leukemia virus RT, Invitrogen). Reverse transcription conditions for each cDNA amplification were 65 °C for 5 min, 37 °C for 52 min, and 70 °C for 15 min. Real-time RT-PCR was carried out using the 7500 Real-time PCR System (Applied Biosystems) and SYBR Premix Ex Taq (TaKaRa). The primers used for the PCR are listed in Table 2.

**Table 2:** Primers used for the quantitative real-time RT-PCR analysis

Gene name	Sequence (5’–3’)
β*-actin*	Sense: TCTTGGGTATGGAGTCCTG Antisense: TAGAAGCATTTGCGGTGG
*FGA*	Sense: GCAGAACAGCATCCAGGAGCAGG Antisense: TCCACCTGGTAATCAAAACTTCTAGCAC
*CA2*	Sense: CACTGGCACGAGCACTTC Antisense: ACTTCACGCCATCCACAGT
*KRT7*	Sense: CTGGACGGGTTGTTAAAT Antisense: TCCGCTTCGTAGAGAGAT
*GRTP1*	Sense: GGCTGCTCCAACGCCCACTT Antisense: CGCAACGCCTTCTGCTCTTT
*MnSOD*	Sense: CTGACCTGCCCTACGACT Antisense: TGGTATGATTGATATGACCC
*LOC429524*	Sense: GGAGGAAATGCGGCGCTTAG Antisense: GGCTGGACGAGACGCTGTTGA
*ATP5A1*	Sense: CAGTTTGGGTCTGATTTGG Antisense: AGCTTAGCTTCCGTCTGG
*FKBP4*	Sense: TACCTCCCAATGCTACGC Antisense: CCTTCGCCTTTCTTACGG
*GOT1*	Sense: GCACAGACCCTACTCCAGAC Antisense: AAGCCCTCGGAGACAAAG
*LMNA*	Sense: GGGGAACTGGCAGGTGAAGC Antisense: CCTCGTCGTCGTCGTTGATG
*PTGDS*	Sense: CCGAGGTCTTTTGTTTG Antisense: AGGAGGGGGACTTTGATG
*HSP*β*1*	Sense: CAAACACGAGGAGAAACA Antisense: CGTTTATTCAAGGCACTG
*Apo A-I*	Sense: TCCGCTTCGTAGAGAGATGTG Antisense: TCAGCGTGTCCAGGTTGTC

β*-actin* acts as internal control; *FGA* encodes the fibrinogen alpha chain, *CA2* encodes carbonic anhydrase II, *Otokeratin* encodes the cytokeratin otokeratin protein, *GRTP1* was predicted to encode the growth hormone-regulated TBC protein 1 protein, *MnSOD* encodes the MnSOD protein, *LOC429524* was predicted to encode a transcription factor 24-like protein, *ATP5A1* encodes the ATP synthase subunit alpha protein, *FKBP4* encodes the PPIase FKBP4 protein, *GOT1* encodes the aspartate aminotransferase 1 protein, *LMNA* encodes the lamin-A protein, *PTGDS* encodes the prostaglandin-H2 D-isomerase precursor protein, *HSP*β*1* encodes the HSPβ1 protein, *Apo A-I* encodes the Apo A-I protein

### Statistical analysis

All results were expressed as mean ± SD and analyzed by student’s *t*-test. Statistical analysis was performed using Prism 5.0 software (GraphPad Software Inc.). *P* < 0.05 was considered to be statistically significant.

## Results

### Differentially expressed proteins between the fat and lean lines of broilers

**Fig. 1:**
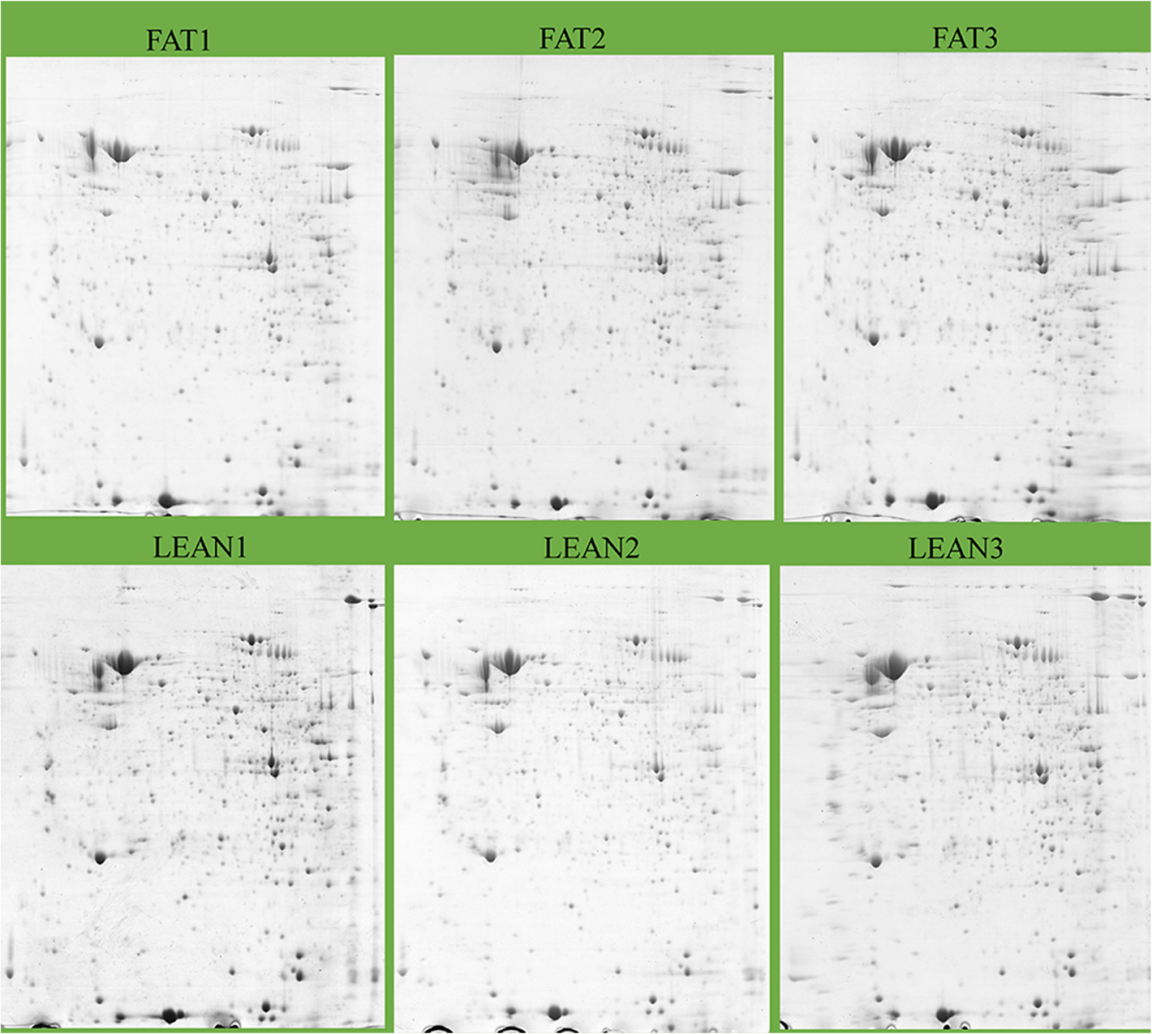
2DE protein profiles of the 4-week-old abdominal adipose tissues of fat and lean broilers. The top three panels represent three biological replicates of the fat broilers, and the bottom three panels represent three biological replicates of lean broilers. These six gels were analyzed by Image Master 2D Platinum 6.0 software (GE Healthcare), and differentially expressed proteins were identified

**Fig. 2:**
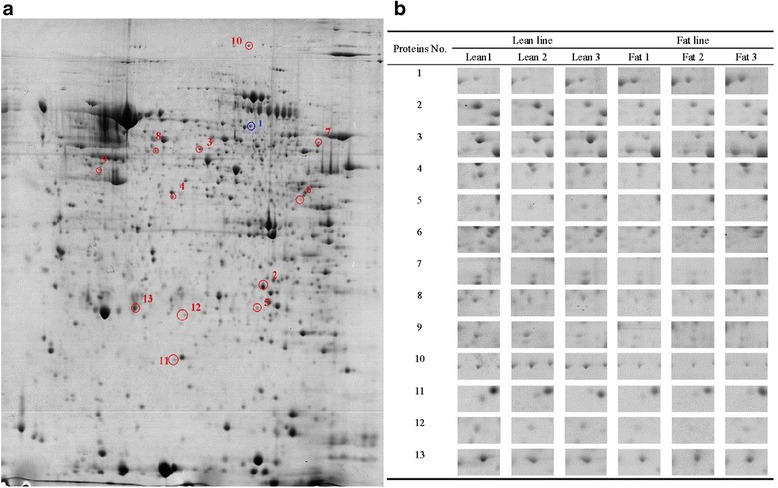
Protein features showing different expression levels in abdominal adipose tissues of fat and lean broilers. **a** A representative image of 2DE gels of the 4-week-old abdominal adipose tissue of broilers. Differentially expressed proteins are circled. The blue circle indicates the protein spot, which was down-regulated in lean broilers. The red circles indicate the protein spots, which were up-regulated in lean broilers. Only 1 protein spot (fibrinogen alpha-E subunit) was down-regulated in lean broilers. **b** Zoom-in images of 13 differentially expressed proteins

Following staining with coomassie blue brilliant G250, the well-resolved 2DE gels were obtained, which are displayed in Fig. 1. We detected 884 ± 12 well-stained, clearly-delineated protein spots per gel (six gels) using Image Master 2D Platinum 6.0 software (GE Healthcare) and most spots were distributed mainly in range of pH 3-10. Quantitative image analysis of three biological replicates of each line revealed that a total of 13 protein spots showed a more than 2-fold difference (*P* <0.05) between the fat and lean broilers. Of these, 12 protein spots were up-regulated and 1 protein spot was down-regulated in the lean birds compared to fat birds (Fig. 2a). The magnification of these 13 protein spots were displayed in Fig. 2b. These 13 differentially expressed protein spots were excised, digested in gel with trypsin and identified by MALDI-TOF-MS. All of the 13 protein spots were identified. The names of the identified proteins, their accession number, expression fold changes between the fat and lean broilers, and other information are shown in Table 3.

**Table 3:** Features of the 13 differentially expressed proteins identified by MALDI-TOF-M

No.	Protein name	Accession number	Fold change^a^	*P-*value	Variation^b^	Mascot score	MW (kDa)	PI	SC^c^ (%)	SL^d^	Biological process
1	Fibrinogen alpha chain	gi|1706798	−3.41	0.025	0.510	204	83.1	5.69	45	S	Signal transduction
2	Carbonic anhydrase II	gi|833606	2.05	0.005	0.595	165	28.8	6.51	68	C	Signal transduction
3	Cytokeratin otokeratin	gi|45384378	2.06	0.002	0.935	264	53.8	5.97	84	NR	Cytoskeleton
4	Predicted: growth Hormone-regulated TBC protein 1	gi|363729049	2.06	0.001	0.273	349	29.5	6.32	62	NR	NR
5	Superoxide dismutase [Mn]	gi|45383702	2.07	0.035	0.468	127	25.1	8.60	48	M	Antioxidant
6	Predicted: transcription factor 24-like	gi|363730972	2.13	0.045	0.855	284	32.9	11.30	71	N	NR
7	ATP synthase subunit alpha	gi|45383566	2.19	0.030	0.595	196	60.1	9.29	48	M	Energy conversion
8	Peptidyl-prolyl cis-trans isomerase FKBP4	gi|57525441	2.20	0.016	0.950	290	51.4	5.34	64	C,N	Antioxidant
9	Aspartate aminotransferase	gi|45384348	2.26	0.003	0.496	164	46.1	8.22	43	C	Amino acid metabolism
10	Lamin-A	gi|45384214	2.30	0.001	1.710	241	73.3	6.50	54	N	Cytoskeleton
11	Prostaglandin-H2 D-isomerase precursor	gi|45383612	2.60	0.010	0.683	155	20.8	6.30	41	C	Lipid metabolism
12	Heat shock protein beta-1	gi|45384222	2.74	0.019	0.518	193	21.6	5.77	37	C	Antioxidant
13	Apolipoprotein AI	gi|227016	2.96	0.039	0.409	253	28.7	5.54	80	S	Lipid metabolism

^a^Fold change: averge relative volume ratio (lean broilers vs. fat broilers)

^b^Variation: Standard deviation

^c^SC: Sequence coverage

^d^SL: Subcellular location. *S* secreted, *C* cytoplasm, *NR* not reported, *M* mitochondrion., N nucleus

### Western blot analysis

**Fig. 3:**
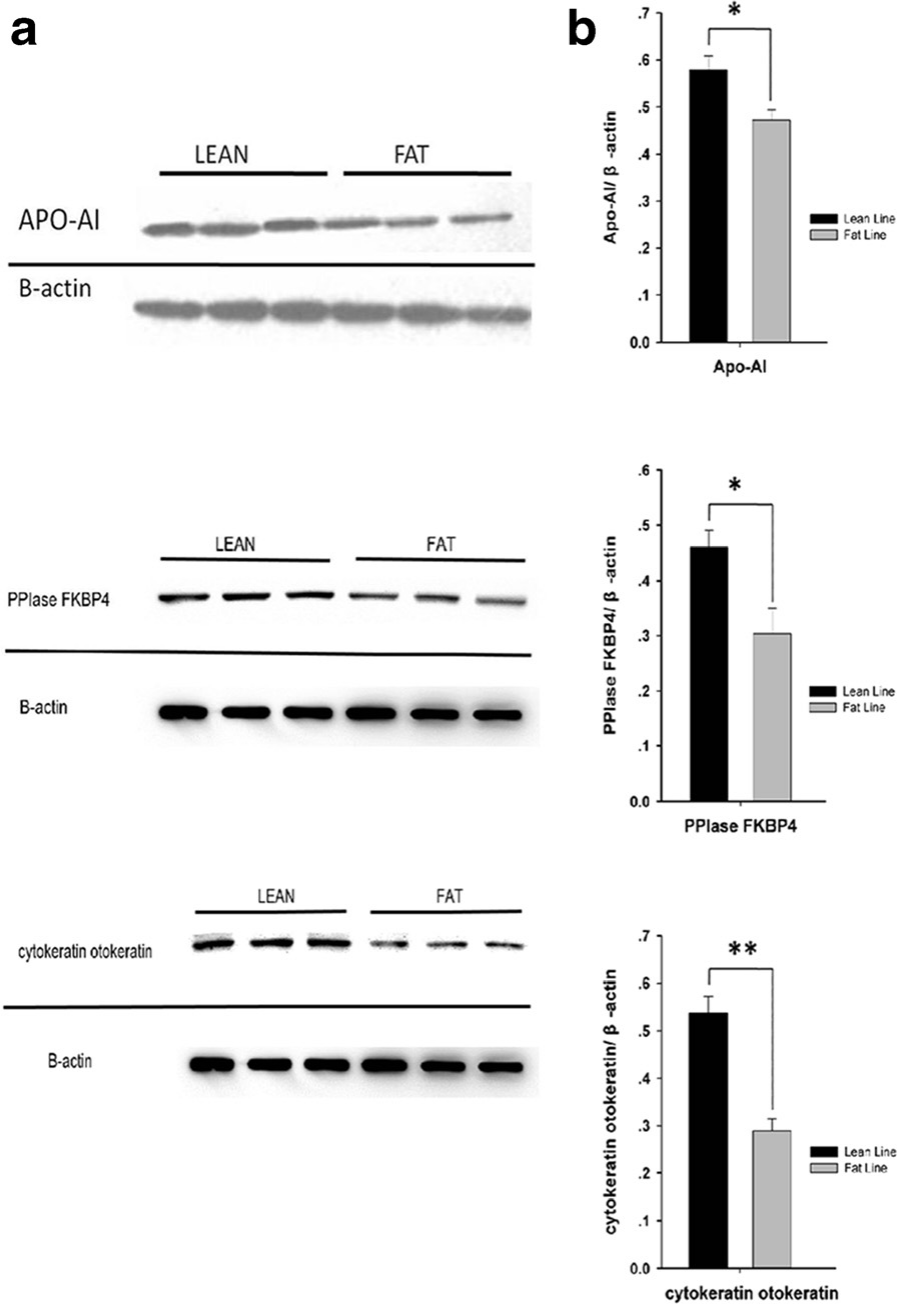
Western blot analysis of three proteins expression in the 4-week-old abdominal adipose tissues of fat and lean broilers. **a** Western blot of three proteins in abdominal adipose tissues of lean and fat broilers. **b** Western blot quantitation of three proteins in abdominal adipose tissues of lean and fat broilers. The expression levels of Apo A-I, PPIase FKBP4 and cytokeratin otokeratin were significantly higher in the lean birds than in fat birds. *P <0.05 or **P <0.01

To verify the differential expression of individual proteins between the fat and lean broilers, we performed western blot analysis. Apo A-I, PPIase FKBP4 and cytokeratin otokeratin protein expression were verified using western blot. As shown in Fig. 3, the expression of abdominal adipose tissue Apo A-I, PPIase FKBP4 and cytokeratin otokeratin protein were significantly higher in the lean birds compared with in the fat birds (*P* <0.05 or *P* <0.01).

### Real-time RT-PCR analysis

**Fig. 4:**
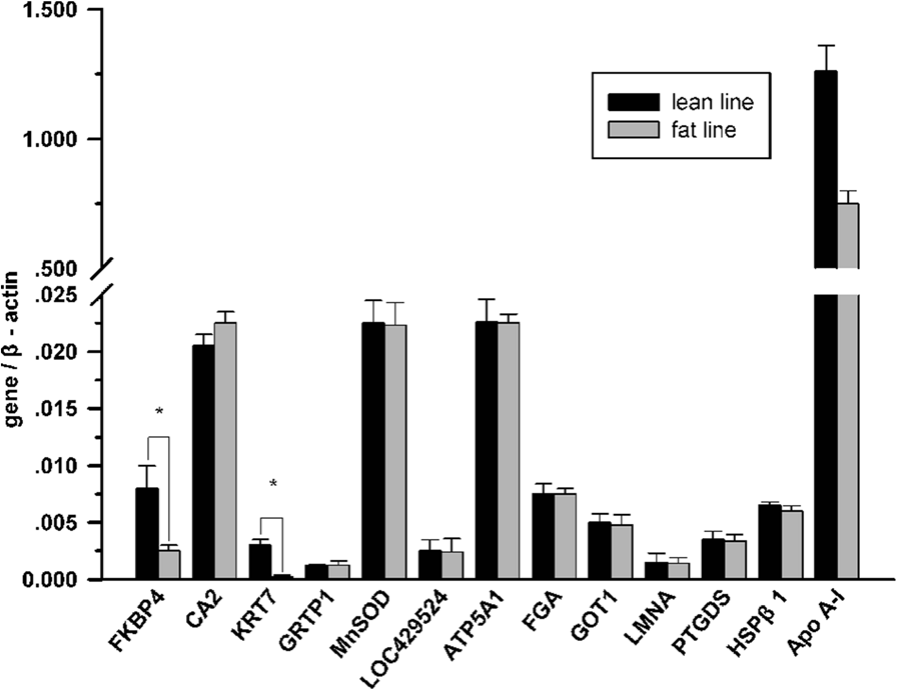
Quantitative real-time RT-PCR analysis of the 13 differentially expressed proteins in the 4-week-old abdominal adipose tissues between lean and fat lines. Only PPIase FKBP4 and cytokeratin otokeratin were significantly differentially expressed at the mRNA level between fat and lean broilers. *P <0.05

We used quantitative real-time RT-PCR to compare the mRNA expression levels of the genes corresponding to 13 differentially expressed proteins between fat and lean birds in abdominal adipose tissues. Surprisingly, the results showed that only two of these transcripts, PPIase FKBP4 and cytokeratin otokeratin, were significantly differentially expressed between fat and lean birds in abdominal adipose tissues (*P* <0.05). The other 11 differentially expressed proteins were not found to be differentially expressed at the mRNA level between the fat and lean lines (Fig. 4). The results are inconsistent with the results of the proteomic analysis.

## Discussion

The NEAUHLF provides an unique experimental model to study growth and development of chicken adipose tissue. In the present study, we identified thirteen differentially expressed proteins in abdominal adipose tissue between fat and lean lines of NEAUHLF at 4 weeks of age. The discovery of these differentially expressed proteins between the NEAUHLF fat and lean broiler lines may provide useful clues for understanding the molecular mechanism of broiler abdominal fat deposition.

Based on the biological process in which they are involved, these differentially expressed proteins could be classified into six categories: lipid metabolism (Apo A-I and prostaglandin-H2 D-isomerase precursor), amino acid metabolism (aspartate aminotransferase), signal transduction (fibrinogen alpha chain and CA-II), energy conversion (ATP synthase subunit alpha), antioxidant (HSPβ1, PPIase FKBP4 and MnSOD), and cytoskeleton (LMNA and cytokeratin otokeratin).

Apo A-I is an important lipid-binding protein, and is the major constituent of high-density lipoprotein (HDL) cholesterol [16]. It plays important roles in preventing lipid accumulation in tissues and in maintaining cholesterol dynamic balance [17, 18]. Genetic deficiency in Apo A-I has been associated with excessive cholesterol accumulation in human and poultry [19–21]. Association studies showed that a single nucleotide polymorphism (SNP) upstream of the ATG initiation codon of the chicken Apo A-I gene was associated with abdominal fat weight and abdominal fat percentage [22]. Our previous proteomic analysis results showed that Apo A-I protein was down-regulated in the abdominal adipose tissue of fat broilers compared with the lean broilers at 7 weeks of age [8]. In the present study, we also observed that Apo A-I was down-regulated in abdominal adipose tissue of the fat broilers. Taken together, these data suggest that the differential protein expression of Apo A-I in the two divergently selected lines may be partially responsible for the difference in abdominal fat deposition between the two broiler lines.

Prostaglandin-H2 D-isomerase precursor is an essential enzyme in arachidonic acid metabolism. It catalyzes the conversion of prostaglandin-H2 to prostaglandin-D2 [23]. One of the dehydration products of prostaglandin-D2 is 15-deoxy-Δ [12, 14]-prostaglandin J2 (15d-PGJ2) [24], which binds directly to peroxisome proliferator-activated receptor γ (PPARγ) and promotes efficient adipocyte differentiation [25]. These findings, combined with the results reported here, suggest that the differential expression of prostaglandin-H2 D-isomerase precursor may contribute to the phenotype difference between the fat and lean broiler lines.

Aspartate aminotransferase 1 is involved in adipocyte glyceroneogenesis, which controls fatty acid homeostasis by promoting glycerol 3-phosphate formation for fatty acid re-esterification when the supply of glucose is reduced [26]. The expression of aspartate aminotransferase 1 is specifically induced by glucose deprivation and rosiglitazone in adipocytes, but it is not directly regulated by PPARγ [27]. In the present study, aspartate aminotransferase 1 was more highly expressed in the lean chickens than in the fat chickens. The differential expression of aspartate aminotransferase 1 may reflect the difference in adipocyte glyceroneogenesis in the two chicken lines.

Fibrinogen alpha chain and CA-II are involved in signal transduction. Fibrinogen alpha chain is an important component of fibrinogen [28]. Kim et al. found that plasma fibrinogen was significantly higher in obese groups compared with in non-obese groups using 2DE and MS, and proposed plasma fibrinogen as a new biomarker of obesity [29]. Fibrinogen is regulated by interleukin-6 (IL-6) in adipose tissue and adipose tissue IL-6 expression was shown to be positively correlated with obesity. IL-6 was found to be the major regulator of fibrinogen, and stimulated fibrinogen synthesis [30]. In high fat diet-induced atherosclerosis in rabbits, high levels of IL-6 and fibrinogen were detected in the plasma [31]. In the present study, we also observed that fibrinogen alpha chain was up-regulated in the abdominal adipose tissue of the fat birds. Taken together, our data suggest fibrinogen alpha chain is involved in broiler fat deposition. Carbonic anhydrases (CAs) are a family of zinc metalloenzymes [32], which is critical to the entire process of fatty acid biosynthesis. A study in human adipose tissue showed that ethoxzolamide, an inhibitor of carbonic anhydrase, significantly reduced the conversion of pyruvate into carbon dioxide, glyceride-glycerol, and fatty acids [33]. In the present study, CA-II was more highly expressed in the lean broilers than in the fat broilers, which is not in agreement with the human study results. This difference may be due to differences in fatty acid synthesis between mammals and birds. In mammals, adipose tissue is one of the major sites for fatty acid synthesis, whereas in birds, adipose tissue is not the major site for fatty acid synthesis, as most of the fatty acid synthesis in birds occurs in the liver.

ATP synthase is responsible for the synthesis of ATP from ADP and inorganic phosphate. Its alpha subunit is essential for its activity and mitochondrial membrane structure [34]. In the present study, ATP synthase subunit alpha was down-regulated in the abdominal adipose tissue of fat birds compared to lean birds. This differential expression may reflect the difference in energy consumption between the fat and lean chicken lines.

Antioxidant enzymes play important roles in oxidative stress resistance. Animals have a complex network of antioxidant proteins that work together to prevent oxidative damage to cellular components such as proteins and lipids [35]. Antioxidants either prevent reactive species from being formed, or remove them before they damage cell components [36]. Adipogenesis is involved in adipocyte hypertrophy and hyperplasia. In human and mouse, adipocyte hypertrophy is correlated with increased oxidant stress and low-grade inflammation, and both are linked to disturbed cellular redox [37]. In the present study, the antioxidant proteins (HSPβ1, PPIase FKBP4 and MnSOD) were differentially expressed between the fat and lean broilers, suggesting that adipose oxidative stress is different in the two chicken lines.

Two cytoskeleton proteins, LMNA and cytokeratin otokeratin, were found to be differentially expressed in adipose tissue between the fat and lean broiler lines in this study. LMNA plays a role in maintaining nuclear stability and chromatin structure [38]. Mutations in the *LMNA* gene were associated with familial partial lipodystrophy [39]. Further studies have shown that LMNA interacts with the adipocyte differentiation factor, sterol regulatory element-binding protein 1 (SREBP-1), and that the reduced binding of LMNA to SREBP1 may be the cause of the familial partial lipodystrophy [40]. Another cytoskeleton, cytokeratin otokeratin, was first detected in the tegmentum vasculosum in chicken [41]. In the present study, we observed that cytokeratin otokeratin was up-regulated in abdominal adipose tissue of the lean birds compared to the fat birds at 4 weeks of age, consistent with our previous proteomic study of adipose tissue in the same two lines at 7 weeks of age [8]. Taken together, the differential protein expression of LMNA and cytokeratin otokeratin suggests that these two proteins are involved in chicken fat deposition.

In addition to the 11 proteins discussed above, transcription factor 24-like and growth hormone-regulated TBC protein 1 were identified by automated computational analysis. The functions of these two predicted proteins remain to be investigated.

It is noteworthy that in the present study, of these thirteen differentially expressed proteins, only two (PPIase FKBP4 and cytokeratin otokeratin) showed consistent expression results at both the mRNA and protein levels. Poor correlation between protein and mRNA levels has been reported in several genomic, transcriptomic and proteomic studies [42, 43]. There are several possible explanations for this poor correlation. One possible explanation is the complicated and varied post-transcriptional gene regulatory mechanisms, for example, microRNAs can inhibit protein synthesis either by repressing translation or by inducing mRNA degradation [44, 45]. Another possible explanation is that in vivo protein half-lives may differ substantially, and the protein half-lives can vary under different conditions [46]. A third possible explanation is our study’s limitation. In the present study, due to the experimental cost, we used a small number (*n* = 3) of biological replicates of the lean and fat broiler lines. Despite the limitation, our study provides the potentially differentially expressed proteins in abdominal adipose tissue between lean and fat broilers.

## Conclusions

In the study, we identified 13 proteins that were differentially expressed in abdominal adipose tissue between the fat and lean broiler lines. Of these proteins, one protein (fibrinogen alpha chain) was more highly expressed in fat broilers, while the other 12 proteins were more highly expressed in lean broilers. All or some of these differentially expressed proteins may be responsible for the phenotype difference between the fat and lean broiler lines.

## References

[1] Baéza E, Le Bihan-Duval E (2013). Chicken lines divergent for low or high abdominal fat deposition: a relevant model to study the regulation of energy metabolism. Animal.

[2] Galic S, Oakhill JS, Steinberg GR (2010). Adipose tissue as an endocrine organ. Mol Cell Endocrinol.

[3] Picard FH, Rouvier R, Marche G, Melin JM (1969). Étude de la composition anatomique du poulet. III. – Variabilité de la répartition des parties corporelles dans une souche de type cornish. Genet. Sel. Evol.

[4] Wang H, Li H, Wang Q, Zhang X, Wang S, Wang Y (2007). Profiling of chicken adipose tissue gene expression by genome array. BMC Genomics.

[5] Wang H, Li H, Wang Q, Wang Y, Han H, Shi H (2006). Microarray analysis of adipose tissue gene expression profiles between two chicken breeds. J Biosci.

[6] Larkina TA, Sazanova AL, Fomichev KA, Olu B, Sazanov AA, Malewski T (2011). Expression profiling of candidate genes for abdominal fat mass in domestic chicken Gallus gallus. Genetika.

[7] Ji B, Middleton JL, Ernest B, Saxton AM, Lamont SJ, Campagna SR (2014). Molecular and metabolic profiles suggest that increased lipid catabolism in adipose tissue contributes to leanness in domestic chickens. Physiol Genomics.

[8] Wang D, Wang N, Li N, Li H (2009). Identification of differentially expressed proteins in adipose tissue of divergently selected broilers. Poult Sci.

[9] Guo L, Sun B, Shang Z, Leng L, Wang Y, Wang N (2011). Comparison of adipose tissue cellularity in chicken lines divergently selected for fatness. Poult Sci.

[10] Berg L, Bearse G (1962). Nutrient requirements of poultry. Poult Sci.

[11] Young C, Truman P (2012). Proteins isolated with TRIzol are compatible with two-dimensional electrophoresis and mass spectrometry analyses. Anal Biochem.

[12] Candiano G, Bruschi M, Musante L, Santucci L, Ghiggeri GM, Carnemolla B (2004). Blue silver: a very sensitive colloidal Coomassie G‐250 staining for proteome analysis. Electrophoresis.

[13] Hindré T, Didelot S, Le Pennec J-P, Haras D, Dufour A, Vallée-Réhel K (2003). Bacteriocin detection from whole bacteria by matrix-assisted laser desorption ionization-time of flight mass spectrometry. Appl Environ Microbiol.

[14] Cottrell JS, London U (1999). Probability-based protein identification by searching sequence databases using mass spectrometry data. Electrophoresis.

[15] Koenig T, Menze BH, Kirchner M, Monigatti F, Parker KC, Patterson T (2008). Robust prediction of the MASCOT score for an improved quality assessment in mass spectrometric proteomics. J Proteome Res.

[16] Barbaras R, Puchois P, Fruchart J, Ailhaud G (1987). Cholesterol efflux from cultured adipose cells is mediated by LpA I particles but not by LpA I: A II particles. Biochem Biophys Res Commun.

[17] Zannis VI, Chroni A, Krieger M (2006). Role of apoA-I, ABCA1, LCAT, and SR-BI in the biogenesis of HDL. J Mol Med (Berl).

[18] Zannis VI, Fotakis P, Koukos G, Kardassis D, Ehnholm C, Jauhiainen M (2015). HDL biogenesis, remodeling, and catabolism. Handb Exp Pharmacol.

[19] Rosales C, Patel N, Gillard BK, Yelamanchili D, Yang Y, Courtney HS (2015). Apolipoprotein AI deficiency inhibits serum opacity factor activity against plasma high density lipoprotein via a stabilization mechanism. Biochemistry.

[20] DiDonato JA, Aulak K, Huang Y, Wagner M, Gerstenecker G, Topbas C (2014). Site-specific nitration of apolipoprotein AI at tyrosine 166 is both abundant within human atherosclerotic plaque and dysfunctional. J Biol Chem.

[21] Kiss RS, Ryan RO, Francis GA (2001). Functional similarities of human and chicken apolipoprotein AI: dependence on secondary and tertiary rather than primary structure. Biochim Biophys Acta.

[22] Wang Q, Li H, Li N, Leng L, Wang G, Ao J (2005). Polymorphisms of Apo-AI gene associated with growth and body composition traits in chicken. Acta Vet Zootech Sin.

[23] Joo M, Sadikot RT (2012). PGD Synthase and PGD_2_ in Immune Resposne. Mediators Inflamm.

[24] Zhu F, Wang P, Kontrogianni-Konstantopoulos A, Konstantopoulos K, Prostaglandin PG (2010). D2 and 15-deoxy-Δ12, 14-PGJ2, but not PGE2, mediate shear-induced chondrocyte apoptosis via protein kinase a-dependent regulation of polo-like kinases. Cell Death Differ.

[25] Kliewer SA, Lenhard JM, Willson TM, Patel I, Morris DC, Lehmann JM (1995). A prostaglandin J 2 metabolite binds peroxisome proliferator-activated receptor γ and promotes adipocyte differentiation. Cell.

[26] Plee-Gautier E, Aggerbeck M, Beurton F, Antoine Bnd, Grimal H, Barouki R (1998). Identification of an adipocyte-specific negative glucose response region in the cytosolic aspartate aminotransferase gene. Endocrinology.

[27] Tordjman J, Leroyer S, Chauvet G, Quette J, Chauvet C, Tomkiewicz C (2007). Cytosolic aspartate aminotransferase, a new partner in adipocyte glyceroneogenesis and an atypical target of thiazolidinedione. J Biol Chem.

[28] Grieninger G (2001). Contribution of the αEC Domain to the Structure and Function of Fibrinogen‐420. Ann N Y Acad Sci.

[29] Kim OY, Shin M-J, Moon J, Chung JH (2011). Plasma ceruloplasmin as a biomarker for obesity: a proteomic approach. Clin Biochem.

[30] Lei H, Xu J, Cheng LJ, Guo Q, Deng AM, Li YS (2014). An increase in the cerebral infarction area during fatigue is mediated by il-6 through an induction of fibrinogen synthesis. Clinics (Sao Paulo).

[31] Zhou B, Pan Y, Hu Z, Wang X, Han J, Zhou Q (2012). All-trans-retinoic acid ameliorated high fat diet-induced atherosclerosis in rabbits by inhibiting platelet activation and inflammation. J Biomed Biotechnol.

[32] Supuran CT (2008). Carbonic anhydrases-an overview. Curr Pharm Des.

[33] Mekary RA, Giovannucci E, Willett WC, van Dam RM, Hu FB (2012). Eating patterns and type 2 diabetes risk in men: breakfast omission, eating frequency, and snacking. Am J Clin Nutr.

[34] Baker LA, Watt IN, Runswick MJ, Walker JE, Rubinstein JL (2012). Arrangement of subunits in intact mammalian mitochondrial ATP synthase determined by cryo-EM. Proc Natl Acad Sci.

[35] Vertuani S, Angusti A, Manfredini S (2004). The antioxidants and pro-antioxidants network: an overview. Curr Pharm Des.

[36] Rochette L, Lorin J, Zeller M, Guilland JC, Lorgis L, Cottin Y (2013). Nitric oxide synthase inhibition and oxidative stress in cardiovascular diseases: possible therapeutic targets?. Pharmacol Ther.

[37] Guo W, Li Y, Liang W, Wong S, Apovian C, Kirkland JL (2012). Beta-mecaptoethanol suppresses inflammation and induces adipogenic differentiation in 3T3-F442A murine preadipocytes. PLoS One.

[38] Gonzalez-Suarez I, Gonzalo S (2010). Nurturing the genome: A-type lamins preserve genomic stability. Nucleus.

[39] Shackleton S, Lloyd DJ, Jackson SN, Evans R, Niermeijer MF, Singh BM (2000). LMNA, encoding lamin A/C, is mutated in partial lipodystrophy. Nat Genet.

[40] DubandGoulet I, Woerner S, Gasparini S, Attanda W, Kondé E, TellierLebègue C (2011). Subcellular localization of SREBP1 depends on its interaction with the C-terminal region of wild-type and disease related A-type lamins. Exp Cell Res.

[41] Heller S, Sheane CA, Javed Z, Hudspeth A (1998). Molecular markers for cell types of the inner ear and candidate genes for hearing disorders. Proc Natl Acad Sci.

[42] Tian Q, Stepaniants SB, Mao M, Weng L, Feetham MC, Doyle MJ (2004). Integrated genomic and proteomic analyses of gene expression in mammalian cells. Mol Cell Proteomics.

[43] Schwanhäusser B, Busse D, Li N, Dittmar G, Schuchhardt J, Wolf J (2011). Global quantification of mammalian gene expression control. Nature.

[44] Eulalio A, Huntzinger E, Izaurralde E (2008). Getting to the root of miRNA-mediated gene silencing. Cell.

[45] Filipowicz W, Bhattacharyya SN, Sonenberg N (2008). Mechanisms of post-transcriptional regulation by microRNAs: are the answers in sight?. Nat Rev Genet.

[46] Glickman MH, Ciechanover A (2002). The ubiquitin-proteasome proteolytic pathway: destruction for the sake of construction. Physiol Rev.

